# Spanish Validation of the “User Reported Measure of Care Coordination” Questionnaire for Older People with Complex, Chronic Conditions

**DOI:** 10.3390/ijerph17186608

**Published:** 2020-09-11

**Authors:** Ester Risco, Glòria Sauch, Anna Albero, Nihan Acar-Denizli, Adelaida Zabalegui, Belchin Kostov, Paloma Amil, Albert Alonso, Ana Rios, Jaume Martín, Núria Fabrellas

**Affiliations:** 1 Intermediated Care Hospital Parc Sanitari Pere Virgili, 08035 Barcelona, Spain; erisco@perevirgili.cat; 2 Research Support Unit Catalunya Central, Fundació Institut Universitari per a la Recerca a l’Atenció Primària de Salut Jordi Gol i Gurina (IDIAPJGol), Catalan Health Institute, Catalunya Central, 08272 Sant Fruitós del Bages, Spain; 3 Emergency Department, Hospital Clínic de Barcelona, 08036 Barcelona, Spain; ALBERO@clinic.cat; 4 Department of Statistics, Faculty of Science and Letters, Mimar Sinan Fine Arts University, 34427 Istanbul, Turkey; nihan.acar@msgsu.edu.tr; 5 FEANS, Hospital Clinic de Barcelona, 08036 Barcelona, Spain; AZABALEG@clinic.cat; 6 Primary Healthcare Transversal Research Group, Institut d’Investigacions Biomèdiques August Pi i Sunyer (IDIBAPS), Primary Care Centre Les Corts, Consorci d’Atenció Primària de Salut Barcelona Esquerra (CAPSBE), 08036 Barcelona, Spain; BADRIYAN@clinic.cat; 7 Chronicity Prevention and Care Programme, Health Planning General Directorate, Ministry of Health, Government of Catalonia, 08028 Barcelona, Spain; pamil@gencat.cat; 8 Fundació Clínic per a la Recerca Biomèdica, 08036 Barcelona, Spain; AALONSO@clinic.cat; 9 Catalan Health Institute, 08007 Barcelona, Spain; a.rios@gencat.cat; 10 Research Suport Unit Barcelona, Fundació Institut Universitari per a la Recerca a l’Atenció Primària de Salut Jordi Gol i Gurina (IDIAPJGol), Catalan Health Institute Barcelona, 08025 Barcelona, Spain; jmartinr.bcn.ics@gencat.cat; 11 Department of Public Health, Mental Health and Perinatal Nursing, Universitat de Barcelona, 08907 Barcelona, Spain; nfabrellas@ub.edu

**Keywords:** integrated care, social care, health care, older people, comorbidity, person centered care

## Abstract

Introduction: Older people with complex, chronic conditions often receive insufficient or inefficient care provision, and few instruments are able to measure their perception of care provision. The “User Reported Measure of Care Coordination” instrument has been satisfactorily used to evaluate chronic care provision and integration. The aim of this study is to validate this instrument in Spanish. Methods: The questionnaire was adapted and validated in two phases: translation and cultural adaptation of the questionnaire and psychometric property measurement. Study population were chronic care conditions patients. Results: A total of 332 participants completed test re-test as part of the questionnaire validation process. The final version of the questionnaire had 6 domains: Health and Well-being (D1), Health day to day (D2), Social Services (D3), Planned Care (D4), Urgent Care (D5), and Hospital Care (D6). Cronbach’s alpha for the overall questionnaire was 0.86, indicating good internal consistency. When analyzing each domain, only Planned Care (D4) and Urgent Care (D5) had Cronbach’s Alphas slightly lower than 0.7, although this could be related to the low number of items in each domain. A good temporal stability was observed for the distinct subscales and items, with intraclass correlation coefficients varying from 0.412 to 0.929 (*p* < 0.05). Conclusion: The adapted version of the “User Reported Measure of Care Coordination” into Spanish proved to be a practical tool for use in our daily practice and an efficient instrument for assessment of care coordination in chronic, complex conditions in older people across services and levels of care.

## 1. Introduction

Healthcare provision to aging populations in the current economic slowdown represents a considerable challenge for major European countries, especially when readdressing policies to cover new demands related to health and social needs [1]. People with complex, chronic conditions often receive inadequate care due to poor coordination between health and social care providers [2]. This situation is even worse when dealing with people older than 65 years. Inefficient coordination is a severe limitation when trying to achieve the triple aim of optimizing health system performance: improvement of health outcomes, the patient’s experience, and care cost-effectiveness [3,4].

Many integrated care initiatives responding to multiple health and social care needs have been introduced in European health systems in a variety of contexts [5,6]. Some countries, such as The Netherlands, Germany, or Austria have already worked to improve care coordination since the early 2000′s [2]. The United Kingdom, with extensive experience working towards integration of care, has also developed and evaluated different potential plans to improve chronic care [7,8]. Spain has done so, with numerous actions undertaken and a variety of state-wide and regional strategies targeting different chronic conditions [9,10]. However, the impact of these strategies has hardly been evaluated, with some exceptions, such as the strategy deployed in the Basque Country [11] or the regional implementation carried out in Spain, Norway, and Greece in the context of the NEXES project (Integrated Care Services supported by Information Technologies for chronic patients with obstructive pulmonary disease, cardiac failure, and/or type II diabetes mellitus [12]).

Despite the implementation of healthcare policies and the great commitment of professionals, there are still significant weaknesses in our healthcare system as it stands: organizational and economic fragmentation between different levels of care, lack of integration between social services and healthcare, lack of coordination with family associations, and long waiting lists for diagnostic and surgical procedures.

In the region of Catalonia, Spain, the Government is already working on a new personalized model based on Person Centered Care called Interdepartmental Plan for Social and Healthcare Interaction (PIAISS) 2017–2020 [13]. This new framework contributes to the development of new strategies to improve care integration. Instruments such as the one developed by Nuño-Solinís et al. [9] have shown very good ease-of-use in daily practice, but its application is limited to the organizational level. We need new instruments to measure care coordination across all levels of care, principally when registering the perspectives of the populations [14]. Through this approach, we aim to measure the impact on the health of the population. Moreover, understanding patients’ experiences is a good way to determine whether our strategies meet the expectations of populations in care coordination for chronic, complex conditions [15]. Therefore, there is *a* need for new validated instruments for use with Spanish-speaking population. In order to guarantee the quality of their measurements, it is essential that the instruments should be subjected to a process of validation. This process consists in adapting the instrument culturally to the setting where its psychometric characteristics are to be administered.

The “User Reported Measure of Care Coordination” questionnaire [16] is a tool developed and validated in the United Kingdom to measure the experiences of patients accessing care across organizational and professional boundaries. This tool has the potential to identify weaknesses with the goal of improving care coordination in line with the expectations of the population. The survey provides some information about how well services support patients and help users to achieve their own life goals. This instrument allows evaluation of the provision of care as well the effectiveness of future interventions for care coordination. The questionnaire consists of 46 items, divided into 7 domains: Health and well-being (D1), Health day to day (D2), Social Services (D3), Planned Care (D4), Urgent access to healthcare (D5), Hospital care (D6), and Self-information (D7). The results are expressed in a single overall numeric variable (0–100) that is expressed as a continuous variable, in which the patients with higher scores have a more positive perception.

The aim of this study is the translation and cross-cultural adaptation of the questionnaire “User Reported Measure of Care Coordination” and the analysis of its psychometric properties.

## 2. Methods

### 2.1. Design

Adaptation and validation of the questionnaire were conducted through a descriptive cross-sectional study. This was carried out in two phases: an initial phase in which the translation and cultural adaptation of the questionnaire was performed and a second phase in which the behavior of its psychometric properties was measured. All research procedures used in this study were established in accordance with the Declaration of Helsinki; participants gave their signed informed consent, and the study was approved by the Ethics Committee at Hospital Clinic, Barcelona (HCB/2017/0731).

### 2.2. Translation and Cultural Adaptation of the Questionnaire

Authorization to translate the questionnaire was obtained from the copyright holders. The transcultural adaptation of the questionnaire was performed following the structured procedure described by Beaton et al. [17]. Two translations of the questionnaire from English to Spanish were done by 2 healthcare professionals working separately. A single, consolidated version of both questionnaires was then produced. This version was then back-translated into English by 2 qualified translators. Finally, both versions in English (the original version and the back-translation) were compared and found to contain no significant differences.

Content validity was performed on the pre-final version of the questionnaire by a panel of 8 expert judges, including 2 nurses and 1 physician from a primary care center, 2 social workers from social services and 1 nurse, 1 physician and 1 social worker from a tertiary hospital. Criteria for the selection of the experts were: (a) experience in evidence-based judgement and evidence-based decision-making (research, publications and experience), (b) availability and motivation to participate, and (c) impartiality. Members of the panel of experts were asked to conduct a qualitative evaluation of every item (degree of understanding, agreement with the text) and were also asked to conduct a quantitative assessment of each item following these criteria: (1) competence (items belong to theoretical established factors), (2) clarity (item is easily understood, its semantics and syntactics are suitable), (3) coherence (item is related to the factor being measured), and (4) relevance (item is essential and has to be included). Each point assessed for each item was quantified on a Likert- type scale of 3 points: (1: Agree; 2: Neither agree nor disagree; 3: Disagree). This was done in two rounds by email. In the first round, all suggestions were added to the pre-final version and the second was performed to obtain agreement among all the experts. None of the items was eliminated at this stage. Some items were adapted to our specific context, and some response categories were combined from the experts’ group discussion. The self-information domain (D7) was removed from the validation process, considering that this was part of the sociodemographic data, and it could not be measured. Finally, the remaining 38 items were divided into 6 domains: Health and Well-being (D1) consisting of 6 items, Health Day to Day (D2) consisting of 14 items, Social Services (D3) consisting of 3 items, Planned Care (D4) consisting of 5 items, Urgent Care (D5) consisting of 3 items, and Hospital Care (D6) consisting of 7 items. Scored items were created by converting the individual responses to questions into scores on a scale of 0 to 100, where a score of 100 represents the best possible response, and a score of 0 represents the worst possible response. Where response options were provided that do not have any bearing on performance in terms of patient experience (such as the ‘Don’t know/Can’t remember’ option), the responses were classified as “not applicable” and no score was given. Questions without answers were not considered too. In order to compute the score for domains, a numeric value (0–100) was also established for each domain. According to this method, at least 2 questions should be answered in the Social Services (D3), Planned Care (D4), and Urgent Care (D5) domains; at least 4 questions in the Health and Well-being (D1) and Hospital Care (D6) domains; and at least 10 questions in the *Health day to day* (D2) domain. Again, higher scores mean better perception.

In order to check viability in daily practice, we conducted a pilot test administering the questionnaire to a sample of 18 patients. Patients answered the overall questionnaire and were asked to review the items, identifying words or concepts they did not understand. No relevant modifications were made.

### 2.3. Psychometric Properties

The characteristics of the participants were similar to those from the sample used by Crump et al. (2017) in the development and validation of the original questionnaire. To ensure a representative sample, registered nurses from across Catalonia, divided into 9 health management areas, were involved in data collection. Forty experienced registered nurses working in different primary care centers received specific training and guidelines on eligibility criteria, clinical tests, and instructions for the administration of the questionnaire. The participation of nurses was voluntary, and each nurse had to include a minimum of 5 participants. The registered nurses recruited participants during a routine visit at the health care center or at home. The participants were selected for convenience in accordance with the following inclusion and exclusion criteria. The participants should be older than 65 years old. Multimorbidity or unique condition difficult to manage according to Complex Chronic Patient (PCC) criteria or advanced chronic illness (limited prognosis, palliative orientation, advanced decisions planned) was required. Understanding oral and written Spanish was necessary. People living in long-term care institutions were excluded. All study participants signed informed consent, and the implications of participating in the study were explained through an information sheet.

A total of 40 experienced registered nurses were responsible for collecting data across Catalonia. Although all of them received specific training, some variations could appear on collecting data.

Sample size was calculated following the criteria established by Kline et al. 2010 [18], which recommend a ratio of 5–10 subjects per item.

Data analysis was performed using the R statistical software package V.3.5.0 for Windows. Descriptive statistics of the quantitative variables are presented as mean and standard deviation. Categorical variables were described according to their frequency. The descriptive analysis on the scores obtained in each domain is presented with the mean and standard deviation.

The intraclass correlation coefficient (ICC) was used to study test–retest reliability of each item between two similar assessments with an interval of 7–10 days between measurements.

The internal consistency of the questionnaire was evaluated using Cronbach’s alpha, which was considered acceptable if the value was greater than 0.7. The “alpha if item deleted” index was computed to study the increase or decrease in the sample value of alpha according to the deletion of items. Correlations between the different items and domains were studied using the Spearman correlation coefficient, assuming that the various domains gather distinct information with moderate-low correlation coefficients. Correlation was considered weak when the *r* value was less than 0.3, moderate between 0.3 and 0.6, and strong when greater than 0.6. A *p* value ≤ 0.05 was considered significant.

## 3. Results

### 3.1. Patient Demographics

A total of 332 patients across Catalonia completed the study. The characteristics of these participants are shown in Table 1. The mean age of the study population was 82.1 ± 8.0 years, and 204 (56.4%) participants were women. From the overall sample, 269 (74.3%) participants lived with a family member and 13 (3.6%) with some other carer such as a friend or neighbor. The rest of the participants lived alone. Only 6 participants (1.7%) described their health status as excellent, 75 (20.7%) said it was good, 198 (54.7%) participants defined it as regular, and 83 (22.9%) described it as bad. From the overall sample, 247 (68.2%) participants did not have any help or additional assistance from a private service or institution for their daily care. The most prevalent health problems in our study sample were hypertension (79%), cardiac diseases (51.4%), diabetes (45.4%), and arthrosis or arthritis (15.7%).

### 3.2. Descriptive Analysis

All questions were answered by the patients that participated in the study except those Conditional questions that could be skipped depending on previous answers. Table 2 shows the frequency distribution of the different questionnaire items. If we look at the non-response pattern for the items answered in the questionnaire, *Health and Well-being* (D1), *Health day to day* (D2), and *Urgent Care* (D5) had practically 99.7% of the items answered. Only in *Health Day to Day* (D2) did we find an item that had a lower score (99.7%), and item (Q8) that was 90.9%. In *Social Services* (D3), *Planned Care* (D4), and *Hospital Care* (D6) there was a lower response rate as participants skipped some questions because they were not receiving this specific care. Therefore, the answer on the perception of Social Services, Planned Care, and Hospital Care cannot be described from the population point of view.

### 3.3. Psychometric Properties

When calculating the scores, we left out 6 items (Q1, Q7, Q21, Q24, Q31, and Q32) because they were considered additional information questions only and could not be measured. Once these items were excluded from the analysis, the Cronbach’s alpha for the overall questionnaire remained at 0.86, indicating good internal consistency. When analyzing each domain, only Planned Care (D4) and Urgent Care (D5) had Cronbach’s alphas slightly lower than 0.7, although this could be related to the low number of items in each domain. Furthermore, for Planned Care (D4), the Cronbach’s alpha value suggested eliminating item Q28, moving internal consistency from 0.35 to 0.58. The internal consistency of each domain and correlations across domains are presented in Table 3. The scores calculated in each domain indicated moderate correlations between Health and Well-being (D1) and Health Day to Day (D2) with the subtraction of the domains. The remaining 4 domains were clearly divided into two blocks: Social Services (D3) with Planned Care (D4), and Urgent Care (D5) with Hospital Care (D6). Social Services (D3) and Planned Care (D4) were poorly correlated with Urgent Care (D5) and with Hospital Care (D6). Table 4 represents the intraclass correlation coefficient (ICC) values for test–retest results. A good temporal stability was observed for the distinct subscales and items, with intraclass correlation coefficients varying from 0.412 to 0.929 (*p* < 0.05).

In total, 9 items were excluded when calculating the scores for different dimensions. Items Q1, Q7, Q21, Q24, Q31, and Q32 were excluded as they were not considered appropriate for a score. The aforementioned questions did not evaluate performance but rather the context or background information. Item Q38 was also excluded due to a low response rate (21.8%) as well as item Q8 since it directly depended on item 7, which was also was excluded due to a low response rate. Finally, “Cronbach’s Alpha if item deleted” suggested removing item Q28, raising the score for internal consistency from 0.35 to 0.58. A total of 24 items achieved one or more positive correlations greater than 0.4 with other items in the survey.

## 4. Discussion

In this study, we translated and validated the “User Reported Measured of Care Coordination” questionnaire into Spanish. This tool aims to capture the perceptions of older people with chronic complex conditions accessing care across organizational and professional boundaries, to identify facilitators and barriers to care coordination activities in our context.

Correlation analysis indicated, on one hand, moderate correlations between Health and Well-being (D1) and Health Day to Day (D2) with other domains and, on the other hand, Social Services (D3) moderately correlated with Planned Care (D4) and Urgent Care (D5) moderately correlated with Hospital Care (D6). However, a high non-response rate was found in 3 specific domains, Social Services (D3), Planned Care (D4) and Hospital Care (D6), as these services had not recently been used by many participants in our study. The non-use of Hospital Care (D6) may be due to the health-related circumstances of each individual but access to Social Services (D3) or Planned Care (D4), considering their specific complexity very much influenced by the characteristics of healthcare and social care systems. Poor access to social services, mostly due to economic issues and system fragmentation, could directly affect the health status of the population [18]. A high percentage of the population are not aware of the existence of care planning [6]. The care plan should be a right of the population so that they can cope better with their chronic complex conditions, and this could be ensured through a formal agreement with our patients [19]. Again, there was a high percentage of missing data when studying the temporal stability in Social Services (D3), Planned Care (D4), and Hospital Care (D6) domains, as described in the original article [16]. According to the results on internal consistency, the “alpha if item deleted” index suggested to remove Q28. Authors finally decided to modify the original scale excluding this item. This was the only difference compared with the original instrument.

This instrument is adapted to our Spanish context following current policies to improve health and social care coordination with a variety of specific strategies across the country. Only a few European countries (e.g., Denmark, Portugal, and Ireland) enjoy a system that integrates social and health care horizontally. In most countries (Austria, Belgium, Spain, Italy, United Kingdom, among others), horizontal fragmentation between the social and health sectors accompanies a vertical division of responsibilities in which three levels of administration (national, regional, and local) with different allocation of power [6,20]. Thus, it is important to develop instruments to measure and improve coordination for each specific context [19]. Access, suitability, and financial sustainability as measurement indicators related to care provision are available in most countries. The quality of care coordination in chronic, complex conditions, however, is a multidimensional phenomenon that is difficult to measure; sometimes, the available data are only numeric measurements, and these do not cover the quality of care from population’s point of view. It is, therefore, almost impossible to make comparisons between countries [21].

In terms of the care coordination process, the following factors appear to be important design features. At a personal level, a holistic focus that allows service users and carers to become more functional, independent, and resilient, and to live well by managing conditions in the home environment. At a service level, it is important to encourage multiple referrals to a single-entry point where care coordination can be supported. At a community level, the role of members of the local community should be seen as integral to the caregiving process. At a functional level, effective communication between members of the multidisciplinary team is essential. At an organizational level, effective targeting of service users is required to prioritize care provision. At a system level, integrated health and social care commissioning can support longer-term strategies and provide a greater degree of stability. Therefore, a political narrative that supports person-centered care coordination provides credibility when developing new ways of working [22,23].

Although the need for care coordination is clear, we have seen there are obstacles within most health care systems [24,25]. This must be overcome and has to be considered a priority. Health care systems remain disjointed and processes vary among and between primary care sites and specialty sites [26]. Patients are often unclear about why they are being referred from primary care to a specialist, and what to do after specialist visits. Referral staff deal with many different processes and lost information, which means that care is less efficient.

Addressing patient health from a broader perspective includes addressing the social determinants that influence health outcomes and engaging patients as collaborative partners in their own wellness. Health systems are beginning to recognize this dynamic interplay between an individual’s social needs, healthy lifestyles, and behaviors and their corresponding health status [24,25].

Previous strategies gave us some recommendations to improve care coordination strategies: Ensure patient—and family—centeredness by identifying family needs and goals that can be supported by a care coordinator [24]. Ensure open lines of communication with the designated care coordinator [27]. Develop a shared understanding of community services and local supports for patients and families. Support interprofessional team training on implementing and measuring care coordination initiatives [28]. Use tools and measures to assess the added value of care coordination for patients and families, including patient/family experience as an outcome measure [24,29].

Our study has some limitations. The results on internal consistency should be interpreted with caution as assessing the internal consistency required omitting conditional items or self-information items. Finally, we believe it would have been preferable to determine the correlation with another tool specifically validated for measuring population perception of care coordination. However, this was impossible, since no other tool was available in the Spanish language. Moreover, although the sample was selected from the whole of Catalonia, there was a particular health management area that could not perform data collection due to a new, recently implemented research intervention.

## 5. Conclusions

Successful deployment of care-coordination is a big challenge that should benefit of tools capturing the experiences of patients as they access care across organizational and professional boundaries. The adapted version of the “User Reported Measure of Care Coordination” into Spanish proved to be a practical tool for use in our daily practice and an efficient instrument for the evaluation of care coordination in chronic, complex conditions in older people across services and levels of care.

Further research is required to assess the extent to which survey data offer information that providers and purchasers can use to make specific changes to improve the quality of their services.

## Figures and Tables

**Table 1:** Sample characteristics (n = 362).

Variable	n (%)
Area	
0	121 (33.4%)
1	21 (5.8%)
2	38 (10.5%)
3	70 (19.3%)
4	20 (5.5%)
5	26 (7.2%)
6	26 (7.2%)
7	40 (11%)
Sex (female)	204 (56.4%)
Who they live with	
Alone	80 (22.1%)
With a family member	269 (74.3%)
Other	13 (3.6%)
Age (years), mean ± SD	82.1 ± 8.0
Person who answered the questionnaire	
The person for whom it is intended	138 (38.1%)
A friend or relative of the person for whom it is intended	86 (23.8%)
The person for whom it is intended and the friend or relative.	79 (21.8%)
The person for whom it is intended with the help of a healthcare professional	59 (16.3%)
Extra help or attention from a private service or institution	
Yes, I pay for other services with my money	76 (21%)
Yes, my family pays for extra help for my care	39 (10.8%)
No	247 (68.2%)
In general, how you describe your state of health	
Excellent	6 (1.7%)
Good	75 (20.7%)
Normal	198 (54.7%)
Poor	83 (22.9%)
Illnesses or health problems	
Hypertension	268 (74%)
Diabetes	164 (45.4%)
Heart failure or other heart problems	186 (51.4%)
Renal failure or other kidney problems	129 (35.6%)
Dementia or cognitive impairment	92 (25.4%)
Others	204 (56.4%)
Osteoarthritis	39 (10.8%)
COPD	36 (9.9%)
Osteoporosis	23 (6.4%)
Hypercholesterolemia	22 (6.1%)

The data is shown as a number and a percentage (%) unless otherwise indicated.

**Table 2:** Frequencies of the categories for the items.

Number of Item	N	% of Responses	1	2	3	4	5	6	7	8	9
D1. Your level of Health, Welfare, and Autonomy											
Q1 ^(1)^	362	100	102 (28.2%)	50 (13.8%)	65 (18%)	126 (34.8%)	19 (5.2%)				
Q2 ^(2)^	362	100	103 (28.5%)	125 (34.5%)	129 (35.6%)	5 (1.4%)					
Q3 ^(3)^	362	100	244 (67.4%)	81 (22.4%)	34 (9.4%)	3 (0.8%)					
Q4 ^(4)^	362	100	102 (28.2%)	168 (46.4%)	82 (22.7%)	10 (2.8%)					
Q5 ^(5)^	362	100	138 (38.1%)	132 (36.5%)	49 (13.5%)	43 (11.9%)					
Q6 ^(5)^	362	100	90 (24.9%)	117 (32.3%)	98 (27.1%)	57 (15.7%)					
D2. Monitoring Your Health											
Q7 ^(6)^	362	100	158 (43.7%)	125 (34.6%)	18 (5.1%)	11 (3.1%)	29 (8%)	77 (21.3%)	10 (2.8%)		
Q8 ^(4)^	329	90.9	206 (62.6%)	107 (32.5%)	14 (4.3%)	2 (0.6%)					
Q9 ^(7)^	361	99.7	259 (71.7%)	78 (21.6%)	18 (5%)	6 (1.7%)					
Q10 ^(4)^	361	99.7	238 (65.9%)	94 (26%)	27 (7.5%)	2 (0.6%)					
Q11 ^(8)^	362	100	45 (12.4%)	261 (72.1%)	56 (15.5%)						
Q12 ^(9)^	362	100	29 (8%)	129 (35.6%)	140 (38.7%)	64 (17.7%)					
Q13 ^(5)^	362	100	179 (49.4%)	134 (37%)	33 (9.1%)	16 (4.4%)					
Q14 ^(10)^	362	100	26 (7.2%)	113 (31.2%)	204 (56.4%)	19 (5.2%)					
Q15 ^(7)^	362	100	185 (51.1%)	133 (36.7%)	30 (8.3%)	14 (3.9%)					
Q16 ^(4)^	362	100	167 (46.1%)	155 (42.8%)	32 (8.8%)	8 (2.2%)					
Q17 ^(4)^	362	100	186 (51.4%)	142 (39.2%)	31 (8.6%)	3 (0.8%)					
Q18 ^(11)^	362	100	253 (69.9%)	64 (17.7%)	21 (5.8%)	9 (2.5%)	14 (3.9%)	1 (0.3%)			
Q19 ^(12)^	361	99.7	141 (39.1%)	117 (32.4%)	42 (11.6%)	29 (8%)	32 (8.9%)				
Q20 ^(5)^	362	100	103 (28.5%)	133 (36.7%)	85 (23.5%)	41 (11.3%)					
D3. Social Services											
Q21 ^(13)^	362	100	118 (32.6%)	244 (67.4%)							
Q22 ^(14)^	118	32.6	58 (49.2%)	6 (5.1%)	46 (39%)	8 (6.8%)					
Q23 ^(15)^	118	32.6	60 (50.8%)	28 (23.7%)	21 (17.8%)	1 (0.8%)	8 (6.8%)				
D4. Treatment Plan											
Q24 ^(16)^	362	100	93 (25.7%)	181 (50%)	88 (24.3%)						
Q25 ^(4)^	93	25.7	48 (51.6%)	40 (43%)	4 (4.3%)	1 (1.1%)					
Q26 ^(4)^	93	25.7	35 (37.6%)	52 (55.9%)	6 (6.5%)						
Q27 ^(5)^	174	48.1	51 (29.3%)	67 (38.5%)	10 (5.7%)	46 (26.4%)					
Q28 ^(4)^	360	99.4	187 (51.9%)	104 (28.9%)	36 (10%)	33 (9.2%)					
D5. Emergency Treatment											
Q29 ^(4)^	362	100	165 (45.6%)	161 (44.5%)	31 (8.6%)	5 (1.4%)					
Q30 ^(5)^	362	100	191 (52.8%)	119 (32.9%)	27 (7.5%)	25 (6.9%)					
Q31 ^(17)^	362	100	238 (65.7%)	16 (4.4%)	46 (12.7%)	8 (2.2%)	0	28 (7.7%)	16 (4.5%)	6 (1.7%)	4 (1.1%)
D6. Hospital Care											
Q32 ^(13)^	362	100	177 (48.9%)	185 (51.1%)							
Q33 ^(18)^	177	48.9	69 (39%)	56 (31.6%)	23 (13%)	18 (10.2%)	11 (6.2%)				
Q34 ^(4)^	177	48.9	65 (36.7%)	63 (35.6%)	34 (19.2%)	15 (8.5%)					
Q35 ^(4)^	177	48.9	67 (37.9%)	57 (32.2%)	41 (23.2%)	12 (6.8%)					
Q36 ^(19)^	177	48.9	34 (19.2%)	130 (73.4%)	1 (0.6%)	12 (6.8%)					
Q37 ^(20)^	177	48.9	79 (44.6%)	65 (36.7%)	33 (18.6%)						
Q38 ^(18)^	79	21.8	37 (46.8%)	33 (41.8%)	2 (2.5%)	2 (2.5%)	5 (6.3%)				

Some 15% of respondents took question P7 to be a multiple-choice question. ^1^ (1) Yes, the person who helps me lives with me; (2) Yes, the person who helps me doesn’t live with me; (3) Yes, the person who lives with me helps me as much as individuals who don’t live with me; (4) I don’t need help; (5) I need help, but I don’t receive help from anyone. ^2^ (1) Yes, I can do all the important tasks for myself; (2) I have some difficulty in carrying out some important tasks; (3) I’m not able to carry out tasks which are important for me; (4) I don’t know/I’m not sure. ^3^ (1) I stay in touch as much as I want with the people around me; (2) I have some contact with people, but not enough; (3) I have very little contact and I feel I somewhat lonely; (4) I have no contact with anyone. I feel completely alone. ^4^ (1) Yes, totally; (2) Yes, partially; (3) Disagree; (4) Completely disagree. ^5^ (1) Totally disagree; (2) Partially agree; (3) Disagree; (4) I’m not sure/I disagree. ^6^ (1) Yes, the Primary Care Physician; (2) Yes, the Primary Care Nurse; (3) Yes, the Primary Care social worker; (4) If another healthcare professional, who?; (5) No, I do it myself; (6) No, a family member or care giver does it; (7) I don’t know/I’m not sure. ^7^ (1) Yes, completely; (2) Yes, to some degree; (3) No; (4) I don’t know/I’m not sure. ^8^ (1) Yes; (2) No; (3) I don’t know/I’m not sure. ^9^ (1) Never work together; (2) They sometimes work together; (3) They always work together; (4) I don’t know/I’m not sure. ^10^ (1) None of my needs have been assessed; (2) Some of my needs have been assessed; (3) All of my needs have been assessed; (4) I don’t know/I’m not sure. ^11^ (1) Yes, they are totally involved; (2) Yes, they are quite involved; (3) They are not sufficiently involved; (4) No, not at all involved; (5) There is no family member or care worker involved; (6) I don’t want my family or a care worker to be involved. ^12^ (1) Yes, they receive the help they need; (2) They get help, but not as much as they need; (3) No, they get little or no help; (4) They don’t need/want help; (5) There’s no family member or care worker involved. ^13^ (1) Yes; (2) No. ^14^ (1) Yes, the right frequency; (2) No, I need fewer visits; (3) No, I need more visits; (4) Not applicable. ^15^ (1) Yes, they last the right amount of time; (2) No, I need a little more time; (3) No, I need a lot more time; (4) No, I have longer than I need; (5) Not applicable. ^16^ (1) Yes; (2) No; (3) I don’t know/I can’t remember. ^17^ (1) A family member; (2) A friend or neighbor; (3) Yes, the Primary Care Nurse; (3) Yes, the Primary Care social worker; (4) Yes, another healthcare professional; (5) No, the Primary Care worker; (6) The emergency services (061); (7) Another healthcare professional (not on this list) who?; (8) Another person (not on this list); (9) I don’t contact anyone. ^18^ (1) Yes, totally; (2) Yes, partially; (3) Disagree; (4) Totally disagree; (5) I don’t know. ^19^ (1) Not sufficiently; (2) Sufficiently; (3) Too much; (4) They didn’t provide me with any information. ^20^ (1) Yes; (2) No; (3) I don’t know/I’m not sure.

**Table 3:** Intercorrelation and internal consistency.

Dimensions	Dimensions						N	Mean (SD)	Cronbach’s Alfa
	D1	D2	D3	D4	D5	D6			
Your level of Health, Welfare and Autonomy (D1)	1						342	62.4 (23.3)	0.73
Monitoring Your Health (D2)	0.528 *	1					334	76.1 (16.6)	0.78
Social Services (D3)	0.312 *	0.056	1				109	59.9 (42.0)	0.83
Treatment Plan (D4)	0.454 *	0.283 *	0.360 *	1			91	74.7 (17.9)	0.58
Emergency Care (D5)	0.360 *	0.462 *	0.108	0.184	1		337	76.5 (23.8)	0.62
Hospital Care (D6)	0.347 *	0.368 *	−0.052	0.088	0.404 *	1	174	67.6 (26.3)	0.77

* Spearman correlation coefficients statistically significant at the 0.05 level. SD: Standard Deviation. Mean (SD)—TOTAL: 71.1 (16.3) Cronbach’s Alfa (TOTAL) = 0.86.

**Table 4:** Test–retest reliability.

Item Number	N	ICC_2,1_	*P*-Value *
Your level of Health, Welfare, and Autonomy			
Q2	332	0.823	<0.001
Q3	332	0.805	<0.001
Q4	332	0.810	<0.001
Q5	332	0.789	<0.001
Q6	332	0.733	<0.001
Monitoring Your Health			
Q9	330	0.778	<0.001
Q10	330	0.622	<0.001
Q11	332	0.698	<0.001
Q12	332	0.635	<0.001
Q13	332	0.655	<0.001
Q14	332	0.412	<0.001
Q15	332	0.751	<0.001
Q16	332	0.750	<0.001
Q17	332	0.758	<0.001
Q18	332	0.644	<0.001
Q19	331	0.741	<0.001
Q20	332	0.691	<0.001
Social Services			
Q22	97	0.910	<0.001
Q23	97	0.913	<0.001
Treatment Plan			
Q25	68	0.929	<0.001
Q26	68	0.878	<0.001
Q27	131	0.743	<0.001
Emergency Care			
Q29	332	0.763	<0.001
Q30	332	0.616	<0.001
Hospital care			
Q33	153	0.734	<0.001
Q34	153	0.821	<0.001
Q35	153	0.841	<0.001
Q36	153	0.803	<0.001
Q37	151	0.829	<0.001

ICC_2,1_: Intraclass Correlation Coefficient. * A value of *p* < 0.05 indicates the temporal stability between two consecutive evaluations.
